# Development of a best-practice clinical guideline for the use of bleomycin in the treatment of germ cell tumours in the UK

**DOI:** 10.1038/s41416-018-0300-x

**Published:** 2018-10-25

**Authors:** Robert A. Watson, Hugo De La Peña, Maria T. Tsakok, Johnson Joseph, Sara Stoneham, Jonathan Shamash, Johnathan Joffe, Danish Mazhar, Zoe Traill, Ling-Pei Ho, Sue Brand, Andrew S. Protheroe

**Affiliations:** 1 Department of Oncology, Oxford Cancer and Haematology Centre, Oxford University Hospitals NHS Foundation Trust, Oxford, UK; 2 Department of Radiology, Oxford University Hospitals NHS Foundation Trust, Oxford, UK; 3 University College London Hospitals NHS Foundation Trust, London, UK; 4 Department of Medical Oncology, Barts Health NHS Trust, London, UK; 5 Department of Oncology, Huddersfield Royal Infirmary, Calderdale & Huddersfield NHS Foundation Trust, Huddersfield, UK; 6 Department of Oncology, Addenbrooke’s Hospital, Cambridge University Hospitals NHS Foundation Trust, Cambridge, UK; 7 Oxford Interstitial Lung Disease Service, Oxford Centre for Respiratory Medicine, Oxford University Hospitals NHS Foundation Trust, Oxford, UK; 8 Bristol Testicular Cancer Service, Bristol Haematology and Oncology Centre, University Hospitals Bristol, Bristol, UK

**Keywords:** Germ cell tumours, Chemotherapy, Adverse effects

## Abstract

Bleomycin, a cytotoxic chemotherapy agent, forms a key component of curative regimens for lymphoma and germ cell tumours. It can be associated with severe toxicity, long-term complications and even death in extreme cases. There is a lack of evidence or consensus on how to prevent and monitor bleomycin toxicity. We surveyed 63 germ cell cancer physicians from 32 cancer centres across the UK to understand their approach to using bleomycin. Subsequent guideline development was based upon current practice, best available published evidence and expert consensus. We observed heterogeneity in practice in the following areas: monitoring; route of administration; contraindications to use; baseline and follow-up investigations performed, and advice given to patients. A best-practice clinical guideline for the use of bleomycin in the treatment of germ cell tumours has been developed and includes recommendations regarding baseline investigations, the use of pulmonary function tests, route of administration, monitoring and patient advice. It is likely that existing heterogeneity in clinical practice of bleomycin prescribing has significant economic, safety and patient experience implications. The development of an evidence-based consensus guideline was supported by 93% of survey participants and aims to address these issues and homogenise practice across the UK.

## Background

Bleomycin is a cytotoxic antibiotic with well-known anticancer activity. First discovered in 1966,^1^ FDA approval for Bleomycin was gained in 1973 and it is an important component of the curative treatment regimens for germ cell tumours and lymphomas.^2^ However, bleomycin can be toxic, with potential long-term complications, and in extreme cases can be fatal.^3^ Due to its inclusion in curative regimens, minimising these effects is of utmost importance, but currently there is lack of consensus on how to prevent and monitor bleomycin toxicity.

The most serious complications of bleomycin are pneumonitis (bleomycin-induced pneumonitis–BIP) and pulmonary fibrosis, but other lung pathology includes organising pneumonia and eosinophilic hypersensitivity pneumonitis. Immune-mediated hypersensitivity, with a potential genetic predisposition, is felt to play an important role in the pathogenesis and subsequent fibrosis that occurs in the later stages.^4–8^ In the case of germ cell tumours, bleomycin-induced pneumonitis is estimated to be around 10% and can be life threatening in up to 20% of these cases.^3^ In patients receiving bleomycin for treatment of lymphoma, incidence of pulmonary toxicity has been reported as high as 18%, with 24% mortality of affected cases.^9^ Other side effects from bleomycin include fever, rash, dermatographic pigmentation upon scratching, cutaneous nodules, alopecia and Raynaud’s phenomenon, although skin changes are thought to be unrelated to lung toxicity.

Diagnosis of BIP is made by combining systemic symptoms such as dry cough and shortness of breath with typical radiological changes on high resolution computed tomography (HRCT). Histological findings on biopsies or lavages and abnormal respiratory function tests can provide supportive, but not diagnostic evidence, and are important for exclusion of atypical infection and other pathologies.

The role of pulmonary function tests (PFTs) in bleomycin-associated lung toxicity is controversial. Recent work has shown that abnormal PFTs pre-treatment do not predict the development of toxicity and are only associated with toxicity at the end of treatment.^10^ However, PFTs have long been used as an indicator of toxicity and reduction in certain values may correlate with radiological changes and development of symptoms.^11^ There is currently a lack of consensus regarding the use of bleomycin according to baseline PFTs.^12^

Even though a cumulative dose has been well described as a cause for bleomycin toxicity,^13^ it is still unclear whether bleomycin toxicity is fully dose-dependent and indeed BIP has been reported with lower doses.^14,15^ Severe reactions may be idiosyncratic with a number other factors likely to be important and BIP has been reported years after bleomycin treatment completion.^16,17^ Multiple studies have shown that patients over the age of 40 are at a much increased risk of BIP compared to patients under 40 in both the treatment of germ cell tumours^3^ and lymphoma,^9^ but the reasons remain unclear. Since bleomycin is mainly excreted by the kidneys (70%), concomitant use of cisplatin has been implicated in BIP due to renal toxicity by cisplatin, which may increase the half-life and toxicity of bleomycin.^18,19^

Bleomycin can be given intravenously (IV), intramuscularly (IM) or subcutaneously (SC); there is currently no consensus regarding administration. Typical schedules involve weekly administration of bleomycin as an IV bolus or short IV infusion. While previous work suggested that the route of administration could potentially impact the level of toxicity^20,21^ and association between rapid IV infusion and higher BIP rates has been reported,^22^ the recently-published TE-3 trial—a phase III randomised study—found no significant difference in the incidence of CT assessed lung toxicity in patients receiving a continuous 72-hour infusion of bleomycin vs. patients receiving daily bolus infusions.^10^

In this present study, we document the heterogeneity of bleomycin use among prescribing clinicians across the UK before suggesting a best-practice guideline intended to harmonise local practice and manage toxicity.

## Methods

Twenty-seven Core questions (supplementary information 1) were developed to assess how Bleomycin is used in the UK among prescribing clinicians in a range of cancer centres. The survey was performed to map current practice, describe heterogeneity and provide a rationale for guideline development—not to directly inform the guidelines themselves. All replies were from specialists in the UK who manage patients with testicular cancer and are experienced in the use of chemotherapy including bleomycin.

### Survey

The survey was developed in survey monkey with a link emailed to clinicians. Email addresses were found using databases from the Association of Cancer Physicians (ACP), and several national testicular trials. In total responses were received from 63 individuals from 32 different cancer centres.

### Survey analysis

Responses were collected via survey monkey and downloaded to a spreadsheet. Analysis was performed using Microsoft Excel.

### Guideline development

A best-practice guideline was developed based on a structured review of existing literature. In some areas, suitable literature is lacking and expert opinion, guided by an understanding of current practice, was used. Relevant literature was identified with MEDLINE searches performed independently by two authors (R.W., H.D.L.P.). Multiple iterations of guidelines were produced and refined based on expert consensus.

## Results

### Results of survey

Responses were obtained from 63 individuals from 32 different cancer centres. Of the 63 replies there were 46 from named individuals and 17 remained anonymous. Fifty-one of these were treating adults with testicular cancer, 12 were looking after teenage and young adult patients. Of the 46 named individuals 44 were specialist clinicians and 2 were nurse specialists.

### Route of administration

**Fig. 1:**
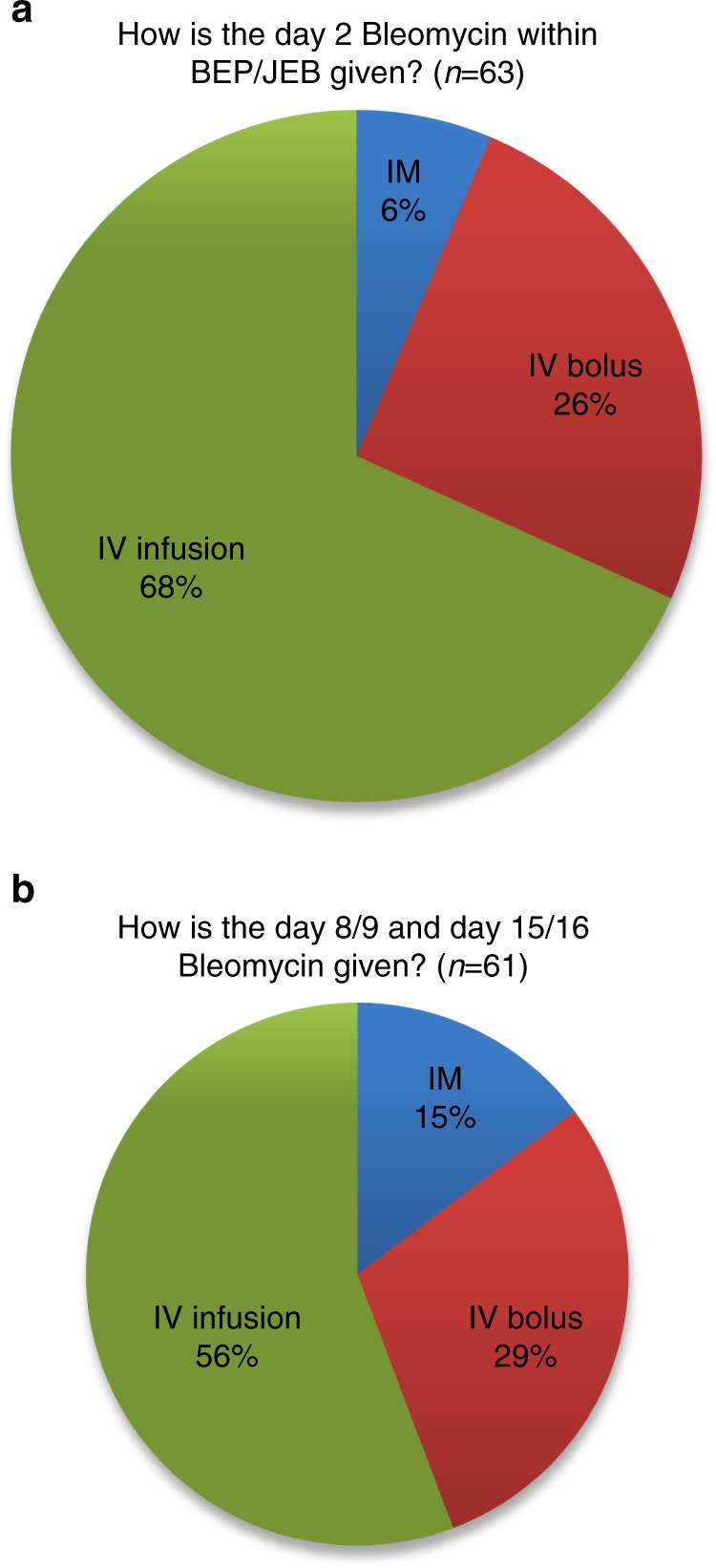
Method of administration of **a** day 2 bleomycin among those surveyed and **b** day 8/9 and 15/16 bleomycin among those surveyed

There was a wide variety in routes of administration of bleomycin both at day 2 and day 8/9 and 15/16. Sixty-eight percent of participants administer day 2 Bleomycin IV as infusion, 26% as IV bolus and 6% as IM injection (Fig. 1a). However, IV infusion rate varied markedly from 30 min up to 18 h. Similarly, IV infusion was the most popular method of administration for day 8/9 and day 15/16, although IM injection and IV bolus were more likely to be used for these subsequent doses (Fig. 1b).

### Use of bleomycin in those with pulmonary risk factors

**Fig. 2:**
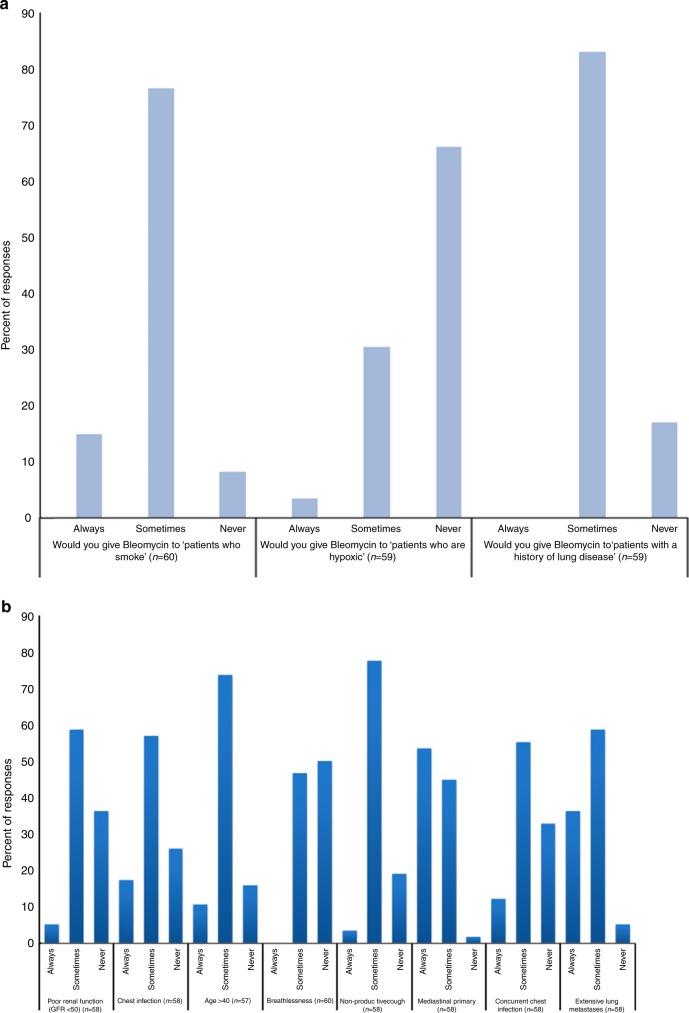
Willingness of participants to use bleomycin in patients with pulmonary risk factors (**a**) and willingness to use bleomycin in those with pre-existing co-morbidities (**b**)

Fifteen percent of participants would use bleomycin in smokers, only 8% of participants would not and the majority (77%) would consider using it depending on individual circumstances. In hypoxic patients, 3% of participants would use bleomycin, 66% would never use it and 31% would consider it depending on individual patient circumstances. Regarding patients with history of lung disease, 17% of participants would never use bleomycin whereas 83% would consider using it (Fig. 2a). In those who would consider using bleomycin in some circumstances, the reasons for using it varied greatly with some suggesting they would be guided by pulmonary function tests, others basing their decision on smoking pack year history.

### Use of bleomycin in those with pre-existing co-morbidities

Five percent of participants would use bleomycin in patients with poor renal function (eGFR < 50), 36% would never use it and 59% would consider it. Eleven percent of participants would use bleomycin in over 40-year olds, 74% would consider it. Fifty percent of participants would avoid bleomycin in patients who are already breathless (Fig. 2b).

### Use of baseline tests prior to bleomycin use

**Fig. 3:**
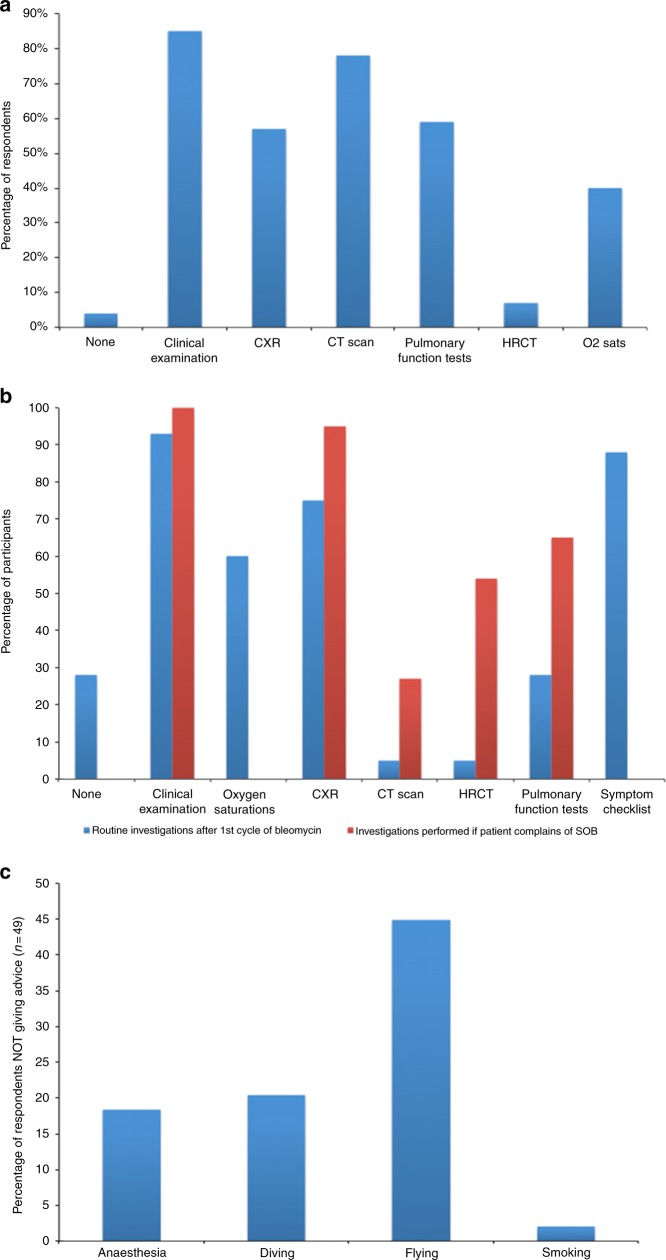
Tests done by participants prior to starting bleomycin (**a**) or routinely following at least one cycle of bleomycin or if the patient reports symptoms (**b**). Frequency of advice not routinely given to patients (**c**)

Regarding baseline test to assess lung function, 85% of participants rely on clinical examination, 78% on staging CT (if available), 59% request pulmonary function tests (PFTs) and 7% request baseline HRCT (Fig. 3). Sixty-four percent of participant complete a ‘toxicity checklist’ before bleomycin is authorised. However, 25% of these lists do not ask about cough and shortness of breath (SOB). Participants seem to agree with giving bleomycin with transfer factor (TLCO) values > 85. Thirty-four percent of participants would use bleomycin with TLCO levels > 75 < 85.

### Follow-up and monitoring

Once bleomycin has been given at least once, 93% participants undertake patient clinical examination, 88% perform a symptom checklist, 74% request chest X-rays (CXRs) and 29% request PFTs prior to the next treatment. If patients complain of shortness of breath (SOB) post-bleomycin, 100% of participants perform clinical examination, 95% request CXR, 65% request PFTs and 54% request HRCT (Fig. 3). If these tests are normal, 70% of participants continue with further bleomycin treatment and 12% stop using it. If a patient develops skin toxicity due to bleomycin, 65% of participants continue its use, whereas 7% stop it.

### Advice to patients

Due to the oxygen-sensitive nature of bleomycin toxicity, patients should avoid inspiring oxygen at high concentrations, including anaesthesia and certain forms of scuba diving. Despite this, 18% of participants responded that they did not routinely give advice regarding anaesthetic and 20% reported that they do not routinely give advice about scuba diving to their patients (Fig. 3c). van Hulst et al.^23^ have published an algorithm to aid in risk stratification of scuba diving post-bleomycin and further advice can be sought from the UK Diving Medical Committee (www.ukdmc.org).

## A best-practice guideline

Based on the significant heterogeneity demonstrated, and the fact that 93% of participants would support it, we have developed a best-practice guideline for the use of bleomycin in germ cell tumours in the UK (Table 1).

**Table 1:** Best-practice guidelines for the use of bleomycin in germ cell tumours in the UK

Stage	Recommendation	Evidence
Baseline investigations	Patients over the age of 40 should receive a baseline CT Thorax prior to commencing bleomycin.	^10^(*Level 1b*)
Pulmonary function tests	Baseline PFTs can be a useful reference in the case of subsequent toxicity and should be considered where possible.	Expert opinion (*Level 5*)
	PFTs should not be used in isolation to aid in a decision as to whether or not to treat with bleomycin.	^10^(*Level 1b*)
	PFTs should not be used as a first-line investigation for suspected lung toxicity.	^10^(*Level 1b*)
	PFTs may aid in the diagnosis of suspected toxicity and may guide management of toxicity. Involvement of a respiratory physician should be considered.	^28^(*Level 1b*) Expert opinion (*Level 5*)
Contraindications to bleomycin	There are no absolute contraindications to use but caution should be exercised with increasing age, significant smoking history, reduced renal function and pre-existing lung disease (in particular pre-existing fibrosis or other symptomatic pathology)	Expert opinion (*Level 5*)
Administration of bleomycin	There is no evidence to support a bolus vs. continuous administration regimen. Typical administration schedules involve a weekly bleomycin bolus or short infusion.	^10^(*Level 1b*)^20^ (*Level 1b*)
Development of bleomycin-related lung toxicity	Cessation of therapy may reverse lung damage and continuing bleomycin therapy may result in worsening toxicity. Continuation in the face of new symptoms should be a consultant decision.	^35^(*Level 1b*)
	Cough is the most sensitive symptom for prediction of toxicity. Dyspnoea is also a significant symptom.	^10^(*Level 1b*)^15^ (*Level 2a*)
	All CT-confirmed diagnoses of bleomycin lung toxicity should be considered for oral Prednisolone (0.5 mg/kg) for 7 days and reduce	^32,33^(*Level 4*) Expert opinion (*Level 5*)
	HRCT chest is indicated if toxicity is suspected with referral to a respiratory physician with an interest in interstitial lung disease.	Expert opinion (*Level 5*)
	Infection should always be considered and treated, and may mimic, coexist with and drive bleomycin-related lung toxicity	Expert opinion (*Level 5*)
	PFTs may have a role in cases of diagnostic uncertainty or high-risk groups (see text)	Expert opinion (*Level 5*)
Post-treatment monitoring	All patients receiving more than 300 units of bleomycin should receive a post-treatment CT scan	^13^(*Level 2a*) Expert opinion *(Level 5)*
	Further investigations should be symptom-led. PFTs are only weakly correlated with increased toxicity at the end of treatment, with DLCO being most significant.	^10^(*Level 1b*)
Symptom monitoring	A ‘toxicity checklist’ should be used before and after every cycle of bleomycin. An example of this can be found in supplementary information 2.	Expert opinion (*Level 5*)
	Renal function should be checked prior to every cycle of treatment.	Expert opinion (*Level 5*)
	Cough is the most important symptom and development of a new cough should trigger further investigation (with HRCT in the first instance).	^10^(*Level 1b*) Expert opinion (*Level 5*)
Advice sheet	Every patient receiving bleomycin should receive a post-treatment advice sheet. An example of this can be found in supplementary information 3.	Expert opinion (*Level 5*)

Levels of evidence are based on the Centre for Evidence-based Medicine Levels of Evidence. http://www.cebm.net/oxford-centre-evidence-based-medicine-levels-evidence-march-2009/

### Baseline imaging

Due to the fact that those of increasing age are at higher risk of developing bleomycin-associated lung toxicity, with those over the age of 30 having 4.8% higher grade ≥ 1 toxicity,^10^ and those over the age of 40 having a twofold increased risk, we recommend performing a baseline CT in all those over the age of 40 receiving bleomycin. Expert consensus felt that a standard CT chest was as helpful as an HRCT for baseline imaging, and all patients are likely to have received this investigation as part of their staging.

### The role of PFTs

The role and importance of PFTs in the monitoring of those receiving bleomycin, and those suspected of toxicity, has long been debated. Some authors have gone as far as advocating regular 3-weekly PFTs^24^ and other studies have positively described the use of this test.^4,25^ However, other work has cast doubt on the utility of PFTs.^26,27^ Commentators disagree on the importance of the different components of PFTs, with diffusion factor (DLCO) felt to be most sensitive but lung capacity more specific.^11,28,29^ Much of the literature pre-dates an era of readily-available cross-sectional imaging, and, on the whole, consists of observational studies or is based on small sample sizes.

A recent prospective phase III randomised study found that PFTs were only weakly correlated with increased toxicity and only at the end of treatment.^10^ We consider this to be the highest level of available evidence and as such we do not recommend performing baseline PFTs with the aim of predicting those likely to develop toxicity. Further, in cases of suspected toxicity (based on symptoms), we consider it superior to investigate using HRCT.

However, we recognise that many practitioners will see value in PFTs and they may be useful for corroborating CT findings or where there is diagnostic uncertainty. Further, PFTs are cheap, non-invasive and quick to perform and are favoured by respiratory physicians. As such, while we do not advocate using PFTs to predict those likely to develop pulmonary toxicity, we recommend performing baseline PFTs where possible, and considering their use (with particular attention paid to the DLCO) as part of the diagnostic process for suspected toxicity, especially in situations of diagnostic doubt.

### Contraindications to bleomycin

We recommend that prescribers recognise that there are no absolute contraindications to use of bleomycin. However, caution should be exercised with increasing age, significant smoking history, reduced renal function and pre-existing lung disease (in particular pre-existing fibrosis or other symptomatic pathology); those with multiple risk factors are more at risk.^13,24,25,30,31^

### Administration of bleomycin

There is no evidence to support a bolus vs. continuous administration regimen for bleomycin. Typical administration schedules involve a weekly bleomycin bolus or short infusion; bleomycin can also be administered intramuscularly. We recognise that choice of regimen will depend on local centre capacity and preferences. However, we recommend that bleomycin should be administered under direct supervision of clinicians familiar with its use and toxicities.

### Development of bleomycin-related lung toxicity

Bleomycin-related lung toxicity should be considered in all patients who develop new respiratory symptoms during systemic treatment with bleomycin. We consider cough to be the most sensitive symptom for predicting toxicity,^10^ however, dyspnoea has also been associated.^15^ In cases of suspected toxicity, HRCT should be the investigation of choice, supported by clinical findings and possibly pulmonary function tests where there is diagnostic uncertainty. Volumetric chest CT can also be used to investigate suspected toxicity. This has the advantage of being able to detect lung metastases and, therefore, update staging, however it carries a higher radiation dose. Therefore, in younger patients where the primary aim is to investigate possible bleomycin toxicity, HRCT may be preferable. CXR has extremely low sensitivity and should not be used as an imaging modality to investigate suspected toxicity.

If lung toxicity is confirmed, cessation of therapy may reverse lung damage and continuing bleomycin therapy may result in worsening toxicity. We recommend that the risks and benefits of stopping or continuing bleomycin treatment are thoroughly considered— including in an MDT setting with both oncologists and radiologists experienced in this condition—and discussed with the patient. To continue with bleomycin in the face of new symptoms of cough or dyspnoea should always be a consultant decision and we consider it safer to omit an individual dose in patients with new cough or dyspnoea than to risk exacerbating toxicity. Such decisions are likely to be guided by the amount and type of changes on CT scan, rapidity of change and the threat of progressive fibrosis and respiratory failure. It should be noted that the changes seen on HRCT can be non-specific and hence the clinical picture needs to be carefully considered.

There is limited evidence to suggest some benefit from high-dose steroids in established cases of acute bleomycin-induced pneumonitis,^32,33^ particularly considering the differential diagnosis that includes atypical pneumonia. In addition, infection should always be considered in cases of possible bleomycin lung toxicity and these can mimic, coexist and drive development of fibrosis. As such we recommend the use of steroids and a low threshold for antimicrobial therapy in acute bleomycin lung toxicity. Steroids are particularly effective in cases of hypersensitivity pneumonitis, but less so in established fibrosis and hence HRCT findings may guide treatment. We also recommend that referral to or discussion with a respiratory physician with an interest in interstitial lung disease is considered in cases of confirmed toxicity.

If doses of bleomycin are omitted due to suspected toxicity, which is subsequently excluded following investigation, these doses can be added into treatment as a weekly dose following completion of the planned treatment cycles. This may prevent complete omission which can result in less favourable outcomes.

### Post-treatment monitoring

All patients receiving more than 300 units of bleomycin should receive a post-treatment CT scan. We acknowledge that this is standard care for those receiving follow-up post-chemotherapy, however the fact that bleomycin was given, and the total dose, should always be included on the radiology request. This is particularly important as pulmonary nodules resulting from toxicity may rarely be mistaken for metastases. Further investigations (e.g., HRCT, pulmonary function tests) should be symptom-led or considered in cases of diagnostic uncertainty. We consider a new cough to be the most important symptom.

### Symptom monitoring

A ‘toxicity checklist’ should be used before and after every cycle of bleomycin. An example of this can be found in supplementary information 2. Cough is the most important symptom and development of a new cough should trigger further investigation (with HRCT in the first instance), as should dyspnoea. Renal function should be checked prior to each new cycle of bleomycin due to increased risk of toxicity with declining renal function.^11^

### Advice sheet

Every patient receiving bleomycin should receive a post-treatment advice sheet. An example of this can be found in supplementary information 3.

## Discussion

This work used a qualitative survey to map the variation in practice in prescribers of bleomycin for germ cell tumours across the UK. Sixty-three participants from 32 different cancer centres responded to the survey. The results show marked heterogeneity in practice in route of administration, willingness to prescribe to those with lung and other co-morbidities, baseline and follow-up investigations performed and advice given to patients. As a result of this, we have produced a best-practice guideline based on current practice, published literature and expert consensus.

### Meaning of the study

Our work has identified the large variation in practice across the UK, even among cancer centres that are in close geographical proximity. It is likely that this was a representation of a lack of a national consensus guideline, meaning that practices have evolved based on local preferences and experience. Many participants responded in the ‘free comment’ section that they have their own local protocols and guidance, particularly surrounding anaesthesia following bleomycin.

Among our participants, 93% agreed that a standardised protocol for the use of bleomycin would be useful. It is, therefore, likely that the heterogeneity of practice observed is due to the lack of an accepted guideline, rather than the unwillingness of clinicians to follow it.

Further, 90% of participants estimated the level of significant bleomycin toxicity in their practice is <10%. In the ‘free comment’ section of the questionnaire, a number of respondents stated that they felt that bleomycin toxicity is a rare and an unusual complication that they infrequently see in their own practice. The uncommon nature of this complication could explain why there is a variation in management and follow-up of these patients and may deepen the need for a standardised approach. However, the incidence of toxicity is likely to be higher than estimated by the respondents—indeed the TE3 study reported that by the end of three cycles, 70.5% of patients had CT evidence of parenchymal changes consistent with BIP (any grade).^10^

It is unclear as to what the effect of the observed heterogeneity is in practice. However, it is likely that there are both patient safety and economic implications. For example 58% of participants request a chest X-ray before starting bleomycin, even though this has an extremely low sensitivity for detecting interstitial lung disease.^34^ An evidence-based best-practice approach to working patients up for bleomycin could potentially save resources and prevent patients undergoing unnecessary tests. As a result of these findings, we have produced a best-practice guideline for the prescribing of bleomycin in germ cell tumours in the UK.

### Strengths and weaknesses of the current study

This work is the first of its kind to map the variation in bleomycin use for germ cell tumours across the UK and propose an evidence-based best-practice guideline for use in this context. We received a high response rate and collected data from 32 different cancer centres, giving a wide spread and range of opinions. Participants were engaged in the work, many left free-text comments and a number were keen on being involved in future developments.

However, we recognise that there are a number of limitations to the work. Regarding the survey, we accept that it is possible that invitations to participate were not received in every case and that there is likely to be some selection bias among participants. In addition, some questions were left unanswered and ‘sometimes’ was often given as a response, which prevents a more in-depth analysis. Finally, this survey was retrospective—so is at risk of recall bias—and is limited to UK practitioners. Regarding the guideline development, the literature was searched for relevant publications, but this was not exhaustive. Consensus was generated from experts (J.J., J.S., D.M., S.S., L.P.H., A.P.) but was not considered at a wider national level. Some of the recommendations are based on expert consensus and there is generally a lack of high-quality randomised or blinded research to support the recommendations and in some areas only the experience and judgement of the practitioner can guide practice. Finally, this current work is solely limited to the use of bleomycin in germ cell tumours. We had hoped to involve expert haematology opinion and circulate the survey to haemato-oncologists but to date this work has not involved haemato-oncologists. Future work regarding the use of bleomycin in UK haematology practice is planned through the expert haemato-oncology groups.

Nevertheless, despite these limitations we are confident that our methods and approach has produced results that can confidently support our conclusion that there is significant heterogeneity in the use of bleomycin for germ cell tumours across the UK and that the guidelines produced represent current best-practice with the available evidence.

### Recommendations for practitioners

The authors strongly support the introduction of this best-practice guideline for the use of bleomycin in germ cell tumours. We accept that some may resist this proposition and there are a wide variety of individual patient factors to take into consideration when deciding whether to proceed with, hold, dose-reduce or stop chemotherapy. We accept this, and it would be up to individual clinicians to use the guideline as they see fit and adapt it to their patients. However, for a relatively rare, but potentially very serious complication, such a wide variation in practice across the UK strongly supports the utility of a consensus guideline developed collectively by the community and available evidence. Ninety-three percent of our participants agreed that such a document would be helpful.

We believe that further research needs to be conducted to more accurately ascertain what ‘best-practice’ looks like and to help further develop and refine the guideline. There is currently a lack of well-designed prospective randomised and blinded studies and while the TE3 trial has provided some evidence, further work is clearly needed. Further, patient involvement is vital in refining and developing information sheets that can give consistent and appropriate advice following bleomycin treatment.

Finally, work to identify those at high-risk of bleomycin toxicity—not just through co-morbidities—but also via genetic, tumoural or phenotypical markers would be of great help in targeting higher intensity monitoring and investigations to those who are most likely to benefit.

## Conclusions

We have demonstrated marked heterogeneity in the clinical practice of bleomycin prescribers in the UK. The adverse effects of such variation are not clear, but we purport that there are significant economic, patient safety and patient experience implications. We have produced a best-practice guideline based on current practice, available published literature and expert consensus.

## Electronic supplementary material

Supplementary information 3

Supplementary information 1

Supplementary information 2

## Data Availability

The questionnaire used is included as supplementary information 1. All other data used in this paper is produced as summary figures
