# Supplementary material for: Development of a best-practice clinical guideline for the use of bleomycin in the treatment of germ cell tumours in the UK
Source: Br J Cancer. 2018 Oct 25;119(9):1044–51. doi: 10.1038/s41416-018-0300-x (PMC6219480; doi:10.1038/s41416-018-0300-x)
Supplement: Supplementary file 1 — Supplementary information 3 [file 41416_2018_300_MOESM1_ESM.docx]

**Supplementary information 3: patient info sheet, bleomycin for germ cell tumours**

Your medical team have given you this information sheet as you will be receiving bleomycin for treatment of your cancer. This sheet contains some important information regarding the risks that bleomycin can pose to the lungs.

**Summary box**

- Bleomycin is an important chemotherapy drug that helps cure germ cell tumours
- Bleomycin can cause lung scarring which in rare cases can be life-threatening
- You must tell your doctor if you develop a new cough or shortness of breath during treatment with bleomycin
- If you have surgery in the future, you should tell the anaesthetist that you have had bleomycin in the past
- If you want to scuba dive in the future, get medical advice beforehand

**What is bleomycin and why do I need it?**

Bleomycin forms an important part of chemotherapy regimens used to treat germ cell tumours (most commonly found as a form of testicular cancer) and lymphoma. Bleomycin is important because research shows that including it can increase the chances that the cancer can be completely eradicated from the body (cured).

**What are the risks of bleomycin?**

Bleomycin has a number of possible side effects which your doctor will go through with you. However, an important possible effect is that bleomycin can cause inflammation (known as interstitial pneumonitis) or scarring (fibrosis) of the lungs. If severe, these can cause difficulty breathing and – in some rare cases – this can be life-threatening.

**What increases the risk of bleomycin to the lungs?**

The risk of developing lung toxicity as a result of bleomycin is increased with increasing age, reduced kidney function or cigarette smoking. People who have had radiotherapy to the lungs, chest or spine can also be at increased risk.

Inhaling a high concentration of oxygen – for example during anaesthetic or in certain forms of scuba diving – can also very much increase the risk of lung toxicity.

**What will my team do to reduce the risk?**

Your medical team will undertake a number of tests before giving you chemotherapy and some of these will be looking particularly at the lungs. These will include CT scans and bedside observations, but your doctor may also request special lung function tests although these are not always required.

**What can I do?**

If you are a smoker, stopping smoking can help reduce the risk. The NHS has a number of stop-smoking tools so speak to your GP or oncologist.

However, it is **very important** that you tell your oncologist if you develop a new cough or shortness of breath during your treatment with bleomycin. Your team should ask you about this before and after every cycle of treatment, but if you notice **any new cough of shortness of breath** you should tell them straight away. If these symptoms develop, it is likely that your oncologist will request further tests – such as CT scans – to investigate it further. This may delay future administrations of bleomycin, but your oncologist will discuss this with you.

If you have surgery in the future, you must tell the anaesthetist that you’ve previously had bleomycin, as this can affect the way they oxygenate the lungs. Some people may choose to wear a medical alert identifier.

If you plan to go scuba diving in the future, seek medical advice first and it may be that you or your doctor needs to contact a dive medicine specialist to get expert advice.
