# Supplementary material for: Development of a best-practice clinical guideline for the use of bleomycin in the treatment of germ cell tumours in the UK
Source: Br J Cancer. 2018 Oct 25;119(9):1044–51. doi: 10.1038/s41416-018-0300-x (PMC6219480; doi:10.1038/s41416-018-0300-x)
Supplement: Supplementary file 2 — Supplementary information 1 [file 41416_2018_300_MOESM2_ESM.docx]

**Supplementary information 1:** Bleomycin survey questions

| **Questions** | **Answer format** |
| --- | --- |
| How do you give your day 2 Bleomycin within BEP/JEB? | Response |
|  | If intravenous over how long is the infusion given? _______ |
| How is the day 8/9 and day 15/16 Bleomycin given? | Response |
|  | If intravenous over how long is the infusion given? |
| Would you give Bleomycin to 'Patients who smoke'? | Response |
|  | If patient smokes, what level of smoking per day would make them ineligible? |
| Would you give Bleomycin to 'Patients who are hypoxic'? | Response |
|  | Please specify hypoxia level at which patients would become ineligible |
| Would you give Bleomycin to 'Patients with a history of lung disease'? | Response |
|  | Please specify lung disease |
| Would you give Bleomycin to 'Patients with poor renal function'> | Response |
|  | Please specify level of renal function at which patients become ineligible |
| Do you use bleomycin containing regimens on the following groups of germ cell tumour patients? | Patients with chest infection |
|  | Patients > 40 |
|  | Patients who are breathless |
|  | Patients with non-productive cough |
|  | Poor renal function with GFR <50 mls/min |
|  | Mediastinal primary |
|  | Concurrent chest infection |
|  | Extensive lung metastases |
|  | Other (please specify) |
| What baseline tests / work up do you organise for patients having Bleomycin (as part of BEP/JEB) to assess lung function? | None |
|  | Clinical Examination |
|  | CXR |
|  | CT Scan |
|  | Lung Function Tests |
|  | High Resolution CT Scan |
|  | Oxygen Saturation Level |
|  | Other (please specify) |
| Do you have a chemotherapy toxicity check-list that is completed before every administration of Bleomycin is authorized? | Response |
| If you do have a check-list does it ask about breathlessness and cough? | Response |
| Would you give Bleomycin with the following parameters from Lung function tests? | TLCO >85 |
|  | TLCO >75<85 |
|  | Comments: |
| What factors reported in a lung function tests would make you NOT give Bleomycin? | Open-Ended Response |
| Do you discuss the results of the lung function tests with a respiratory physician? | Lung Function |
|  | When would you discuss with respiratory physician |
| On subsequent cycles what standard investigations do you do for Bleomycin? | None |
|  | CXR |
|  | Clinical Examination |
|  | Oxygen Saturation |
|  | CT Scan |
|  | Lung Function Tests |
|  | High Resolution CT Scan |
|  | Symptom Check List |
|  | Other (please specify) |
| If a patient complains of mild breathless post first cycle of Bleomycin, what investigations would you complete? | Clinical Examination |
|  | Lung Function Tests |
|  | CT Scan |
|  | CXR |
|  | High Resolution CT Scan |
|  | Other (please specify) |
| If all of the above tests are normal would you continue with Bleomycin if the patient still has mild breathlessness? | Response |
|  | Other (please specify) |
| What factors would make you stop Bleomycin early? | Open-Ended Response |
| If a patient has skin toxicity secondary to Bleomycin, would you stop treatment with bleomycin? | Response |
|  | Other (please specify) |
| How often do you formally assess patients during treatment? | Open-Ended Response |
| What would you estimate as the level of mild Bleomycin toxicity (not requiring intervention) in patients that you see? | Response |
|  | Free text for any comments |
| What would you estimate as the level of significant Bleomycin toxicity in patients that you see? | Response |
|  | Free text for any comments |
| If Bleomycin is stopped what would you do with subsequent cycles of Chemotherapy? | Open-Ended Response |
| What information do you give patients on Bleomycin with regard to the following? | General Anaesthesia |
|  | Diving |
|  | Flying |
|  | Smoking |
| Would you find a standard protocol developed through the clinical studies group for the use of Bleomycin useful? | Response |
| Please add any comments you wish to make regarding the use of Bleomycin in patients with germ cell tumours | Open-Ended Response |
| Are you willing to provide your Bleomycin protocol? - Please tick box if you are willing to send us your protocol for Bleomycin and we will email you. | Response |
| Please provide your Name and Email Address. |  |
