# Supplementary material for: Development of a best-practice clinical guideline for the use of bleomycin in the treatment of germ cell tumours in the UK
Source: Br J Cancer. 2018 Oct 25;119(9):1044–51. doi: 10.1038/s41416-018-0300-x (PMC6219480; doi:10.1038/s41416-018-0300-x)
Supplement: Supplementary file 3 — Supplementary information 2 [file 41416_2018_300_MOESM3_ESM.docx]

Appendix 2: Bleomycin toxicity checklist

Space for patient label

Today’s date:

Date of bleomycin:

Details of chemotherapy regimen (e.g. number of intended cycles; 3- or 5-day BEP etc):

Cycle number and day: Cycle: Day:

Please indicate (circle) if checklist is being completed pre- or post-treatment:

Pre-treatment Post-treatment

|  |  | Consult Medical Team if: |
| --- | --- | --- |
| Pulse |  | > 101 |
| Respiratory Rate |  | > 19 |
| O2 Sats |  | < 94% |

|  | **Yes** | **No** |
| --- | --- | --- |
| Does the patient report new cough?  *New is defined as a cough of date of onset that post-dated the first bleomycin dose administered.* |  |  |
| Does the patient report new shortness of breath?  *New is defined as a cough of date of onset that post-dated the first bleomycin dose administered.* |  |  |
| Does the patient report any other new respiratory symptoms?  *New is defined as a cough of date of onset that post-dated the first bleomycin dose administered.* |  |  |
| Have renal function tests been checked?  *Caution in cases of deteriorating renal function.* |  |  |

**If the answer to any of the above is ‘yes’ please discuss with the Consultant Oncologist in charge of this patient’s care. Consider HRCT before proceeding with any further bleomycin.**
